# The Origin of Abnormal Beta Oscillations in the Parkinsonian Corticobasal Ganglia Circuits

**DOI:** 10.1155/2022/7524066

**Published:** 2022-02-25

**Authors:** Atefeh Asadi, Mojtaba Madadi Asl, Abdol-Hossein Vahabie, Alireza Valizadeh

**Affiliations:** ^1^Department of Physics, Institute for Advanced Studies in Basic Sciences (IASBS), Zanjan, Iran; ^2^School of Electrical and Computer Engineering, College of Engineering, University of Tehran, Tehran, Iran; ^3^Department of Psychology, Faculty of Psychology and Education, University of Tehran, Tehran, Iran

## Abstract

Parkinson's disease (PD) is a neurodegenerative brain disorder associated with motor and nonmotor symptoms. Exaggerated beta band (15–30 Hz) neuronal oscillations are widely observed in corticobasal ganglia (BG) circuits during parkinsonism. Abnormal beta oscillations have been linked to motor symptoms of PD, but their exact relationship is poorly understood. Nevertheless, reduction of beta oscillations can induce therapeutic effects in PD patients. While it is widely believed that the external globus pallidus (GPe) and subthalamic nucleus (STN) are jointly responsible for abnormal rhythmogenesis in the parkinsonian BG, the role of other cortico-BG circuits cannot be ignored. To shed light on the origin of abnormal beta oscillations in PD, here we review changes of neuronal activity observed in experimental PD models and discuss how the cortex and different BG nuclei cooperate to generate and stabilize abnormal beta oscillations during parkinsonism. This may provide further insights into the complex relationship between abnormal beta oscillations and motor dysfunction in PD, which is crucial for potential target-specific therapeutic interventions in PD patients.

## 1. Introduction

**Figure 1:**
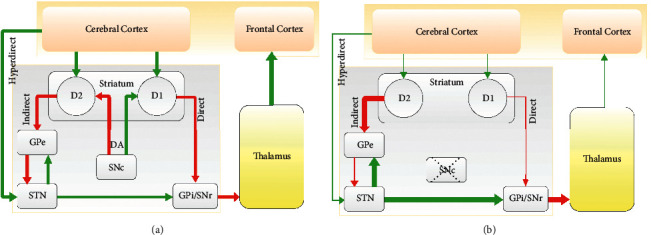
Schematic illustration of the CBGTC loop. The classical model of the BG consists of the opposing direct and indirect pathways. The hyperdirect and DAergic pathways are also shown. (a) CBGTC loop in the normal state. (b) CBGTC loop in the PD state characterized by loss of DA and alteration of inputs. Green and red arrows represent excitatory and inhibitory inputs, respectively. Thickness of arrows indicates the strength of inputs.

Parkinson's disease (PD) is a movement-related brain disorder that is associated with widespread neurodegeneration within the basal ganglia (BG) [1, 2]. BG are a group of subcortical nuclei comprising striatum, globus pallidus (GP), substantia nigra (SN), and subthalamic nucleus (STN). The (dorsal segment of) striatum consists of the caudate and putamen. The caudate is associated with limbic functions, whereas the putamen is related to the motor functions of the striatum. The GP is divided into the external (GPe) and internal (GPi) segments. The SN consists of two parts, i.e., pars compacta (SNc) and pars reticulata (SNr) segments. The SNc is considered as the main source of dopaminergic (DAergic) cells in the BG. The classical model of BG circuitry is schematically shown in Figure 1(a). In this model, the striatum receives direct excitatory input from the cortex and projects to the output nuclei of BG (i.e., GPi/SNr) via two competing pathways: The direct pathway (i.e., striatum ⟶ GPi/SNr) and the indirect pathway (i.e., striatum ⟶ GPe ⟶ STN ⟶ GPi/SNr).

The BG, cortex, and thalamus together form the cortico-BG-thalamo-cortical (CBGTC) loop, where the BG receive inputs from the cerebral cortex and relays them toward the frontal cortex via thalamus (see Figure 1(a)). The CBGTC loop plays a key role in action selection and movement-related tasks, as implicated in several movement disorders such as PD, essential tremor, and Huntington's disease [3]. Structural and functional changes [4–7] within the BG following significant degeneration of DAergic neurons impair normal excitatory-inhibitory input balance toward the BG output nuclei (cf. Figures 1(a) and 1(b)). This leads to the hypoactivity of neurons in the direct pathway, whereas neurons in the indirect pathway become hyperactive, further inhibiting the GPe [8]. Consequently, competition between reduced inhibitory drive from GPe to STN and altered cortex-STN excitation via the hyperdirect pathway [7] leads to the excessive excitation of GPi/SNr. In this way, the inhibitory drive to the thalamocortical pathway is enhanced. This ultimately disturbs the normal function of the CBGTC loop [3].

PD is characterized by several motor and nonmotor symptoms that some of them are linked to neuronal loss, whereas others are attributed to abnormal neuronal activity [2]. In particular, motor symptoms of PD such as tremor, rigidity, akinesia, and bradykinesia are linked to significant degeneration of DAergic neurons in the SNc and their projections to the striatum [9, 10]. DA loss triggers a series of maladaptive or compensatory changes in the BG, ultimately leading to the emergence of pathological dynamics and structure within and between different BG nuclei [10, 11]. Experimentally, alteration in the firing rates of different BG nuclei is widely observed both in animal PD models and PD patients [11]. Apart from some contradictory observations [12, 13], increased firing rates of STN neurons and decreased firing rates of GPe neurons have been confirmed by several PD-related studies [10, 12]. Abnormal neuronal activity is subsequently transmitted to the BG output nuclei and then to thalamus and cortex, which contributes to the symptoms observed in PD.

Emergence of exaggerated beta band (15–30 Hz) neuronal oscillations in the cortex and different BG nuclei, especially in the STN of animal PD models [10] and PD patients [14], is one of the abnormal functional changes following DA loss. Although the hyperactivity of STN neurons [15] and motor symptoms of PD [14] are early phenomena in PD condition, experimental findings suggested that the enhancement of abnormal beta oscillations within the BG may not appear in the early stages of PD. Rather, they may be correlated with the extent of progressive degeneration of nigral DAergic neurons [16]. However, the observation of enhanced beta oscillations in the motor segment of STN in parkinsonian animals [10] and humans [17] suggests that these oscillations may be associated with the motor signs of PD. How exactly these abnormal beta oscillations are linked to the emergence of motor symptoms is still a matter of debate [18].

Yet, the findings of several experimental studies revealed that the reduction of the abnormal beta band oscillatory activity by DA medication therapies [19, 20] or deep brain stimulation (DBS) techniques [21, 22] correlates with clinical improvement in motor symptoms of PD. This implies that although the abnormal beta oscillations may not be the primary cause of motor symptoms, their suppression is strongly correlated with the alleviation of the PD motor symptoms. Typically, it is assumed that the GPe-STN network is responsible for abnormal rhythmogenesis in the parkinsonian BG; however, the role of other nuclei in the appearance of PD-related patterns of neuronal activity deserves more scrutiny. To shed light on the role of corticobasal ganglia circuits in the emergence and stabilization of abnormal beta oscillations in PD condition, here we review the experimentally observed changes of neuronal firing rates and firing patterns as well as local field potential (LFP) recordings within the cortex and each BG nucleus following DA loss. This is of importance since whether or not the abnormal beta oscillations are behind the emergence of motor symptoms, their reduction can induce therapeutic effects both in computational and experimental PD models [21–23]. In this review, we try to elucidate how different dynamical and structural changes in the BG network could have a role in the emergence of parkinsonian beta oscillations. Finally, we briefly discuss the potential role of other mechanisms, such as synaptic plasticity, in shaping physiological and pathological patterns of neuronal activity and synaptic connectivity in the BG.

## 2. Methods

To provide insights into the origin of abnormal beta oscillations in PD, a review of the existing literature was conducted using PubMed. The search was carried out between September 1, 2020, and September 1, 2021, using the search terms “Parkinson,” “beta,” “firing rate,” and “dopamine” combined with the Boolean operators “AND” and “OR.” Experimental studies investigating mechanisms in animals and humans were considered. The titles and abstracts of 645 potentially relevant studies were screened. Then, for abstracts that met inclusion criteria the full texts were retrieved and independently reviewed (56 studies). Additional studies were identified by searching through the references of previously selected papers (29 studies), and 17 more papers were included in the list of the references.

## 3. Neuronal Activity within the Corticobasal Ganglia Circuits

Changes of the neuronal firing rates within the cortex or different BG nuclei in PD patients [24, 25] and animal models [8, 10, 26] have been reported by numerous studies. However, a number of studies argued that functional changes in the BG following DA depletion are associated with changes of the neuronal firing patterns (e.g., spiking vs. bursting), rather than just firing rates [12, 13]. These observations challenge the classical rate-based interpretation of the emergence of PD motor symptoms. Below, we will review changes of neuronal firing rates and firing patterns as well as synchrony between neuronal activities in the normal and PD state observed within the cortex and different BG nuclei in experimental models.

### 3.1. Cortex

**Figure 2:**
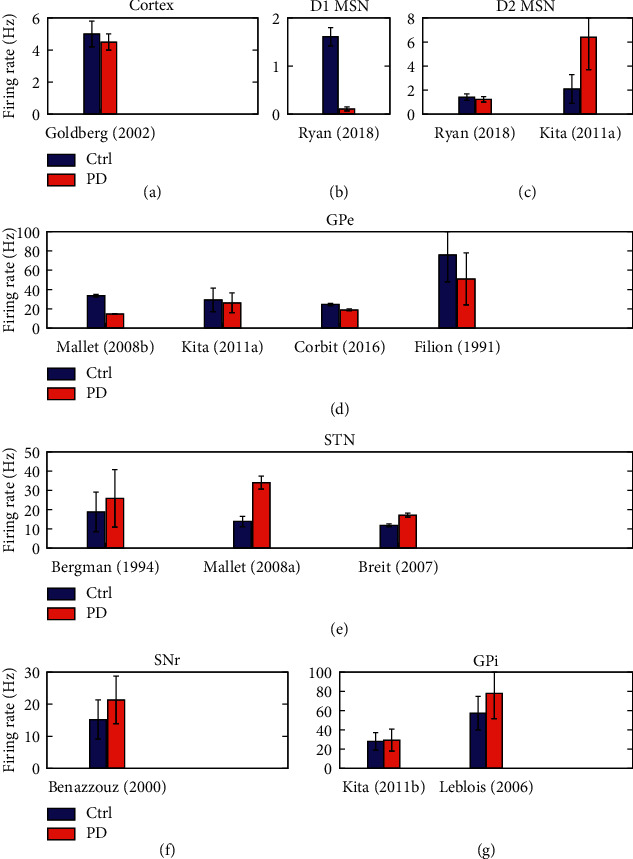
Changes in the neuronal firing rates in corticobasal ganglia circuits (denoted above each panel), which are listed in Table 1, are visualized for a better comparison between their behavior in control (blue) and PD (red) condition.

In the past years, PD studies were mostly devoted to the inspection of neuronal activity changes in the subcortical networks (for a review see Ref. [11]). Hence, neuronal activity within the cortex, specially at the level of single neurons, attracted less attention. A few studies observed no specific changes in the spontaneous activity of neurons in the primary motor cortex (4.5 ± 0.5 Hz, PD, vs. 5.0 ± 0.8 Hz, ctrl) [27] or supplementary cortices [28] of 1-methyl-4-phenyl-1,2,3,6-tetrahydropyridine (MPTP)-treated nonhuman primates (also see Table 1, row 1, and Figure 2(a)). Later, it was suggested that the firing rates of primary motor cortical neurons projecting to the pyramidal tract may be reduced in parkinsonian monkeys [36]. However, the firing rates of primary motor cortical neurons projecting to striatum were relatively unchanged [36].

Intriguingly, the alterations of the neuronal dynamics in the primary motor cortex during parkinsonism is not limited to the firing rate changes, rather the properties of neuronal firing patterns or the level of neuronal synchrony may also be affected. For instance, the percentage of primary motor cortical bursting neurons was increased in a model of MPTP-treated monkeys [27]. Similar observations were made for the cortico-spinal neurons, but not corticostriatal neurons, in another study involving MPTP-treated monkeys [36]. Moreover, the emergence of excessive neuronal synchronization was validated among primary motor cortical neurons in a MPTP-treated nonhuman primate model of PD such that the percentage of strongly synchronized neuron pairs was significantly enhanced (∼41%, PD) compared to the control condition (∼10%, ctrl) [27].

Changes of cortical neuronal activity may be the result of interaction between cortex and other networks in the CBGTC loop. However, the source of these changes may also be traced back to the cerebral cortex itself. In fact, DA depletion may affect the dynamics of the motor cortex and lead to the alteration in the intracortical inhibition [37]. This leads to the remapping of the functional connectivity within the motor cortex and can cause abnormal corticostriatal neuronal synchronization upon movement initiation that may contribute to the motor symptoms of PD [37]. Clinical therapeutic effects induced by cortical stimulation suggest that suppression of this abnormal synchrony can be beneficial in PD patients [38].

### 3.2. Striatum

The striatum is the main input nucleus of the BG. The neurons embedded in the striatum are mostly characterized as the gamma-aminobutyric acid (GABAergic) medium spiny neurons (MSNs). However, the presence of cholinergic interneurons referred to as the fast spiking interneurons (FSIs) or other types of GABAergic interneurons can affect the spiking activity of MSNs. For instance, FSIs receive inputs from the GPe, thalamus, and cerebral cortex and project onto the MSNs. Subpopulations of MSNs are typically classified based on their DA receptor expression (i.e., D1-like vs. D2-like) [39]. The direct pathway is characterized with D1 MSNs, whereas D2 MSNs act in the indirect pathway. In the classical model of the BG, MSNs in the direct and indirect pathways are opposingly modulated by DA so that neuronal activity in the direct pathway is enhanced by the activation of D1 receptors (D1Rs), whereas neuronal activity in the indirect pathway is suppressed by the activation of D2 receptors (D2Rs) [40]. In this way, DA loss can disturb excitation-inhibition balance in these pathways, leading to the appearance of abnormal neuronal activity in PD [15]. In the indirect pathway, the STN receives inhibitory input from GPe. It also receives direct excitatory input from motor-related areas of the cortex via the hyperdirect pathway (see Figure 1(a)), which can further modulate the neuronal activity within the STN [7].

Following DA loss in PD condition, the firing rates of D1R and D2R MSNs are differently modulated. The activity of D1R MSNs in the direct pathway is suppressed in the PD state, whereas the activity of D2R MSNs acting in the indirect pathway is enhanced in the parkinsonian state [34, 41]. However, the experimental findings on the firing rate changes of striatal neurons are contradictory in some cases [12, 13].

Particularly, in 6-hydroxydopamine (6-OHDA)-treated mice, the firing rate of D1R MSNs is suppressed compared with the control condition (0.11 ± 0.04 Hz, PD, vs. 1.61 ± 0.19 Hz, ctrl) [29]. The same study reported that the firing rate of D2R MSNs remains relatively unchanged in the PD state in comparison with the control condition (1.24 ± 0.23 Hz, PD, vs. 1.42 ± 0.28 Hz, ctrl) [29]. On the contrary, a study on the 6-OHDA rat model of PD showed enhanced neuronal firing rates of D2R MSNs compared with the control condition (6.4 ± 2.7 Hz, PD, vs. 2.1 ± 1.2 Hz, ctrl) [12], which is inconsistent with the results reported in Ref. [29] (also see Table 1, rows 2, 3, and Figures 2(b) and 2(c)). Another study in 6-OHDA mice showed that simultaneous blockade of D1Rs and D2Rs decreases the firing rate of the majority of neurons (82.25 ± 13.88%) [42]. Similar effects were observed due to the blockade of D1Rs (89.09 ± 5.84%) and intriguingly also after the blockade of D2Rs (75.84 ± 11.50%) [42].

However, a study on PD patients reported no drastic change in the neuronal firing rates and no locking between spike and beta LFP oscillations in the striatum compared with data obtained in healthy nonhuman primates [13]. Furthermore, the discharge properties of the subpopulations of MSNs (i.e., D1R MSNs or D2R MSNs) were hardly distinguishable from each other, implying no significant discharge change in the pathological state [13]. Such observations challenge the predictions of the rate-based model regarding the alternations of firing rates in striatal neurons following DA depletion and are not consistent with early experimental observations obtained in a number of animal models [12, 42]. Nonetheless, the firing rate changes of striatal neurons may be related to the alterations in corticostriatal transmission, rather than intrinsic changes in the activity of D1R or D2R MSNs [11, 41].

The number of other groups of cells in the striatum is in minority compared with the MSNs and, hence, received less attention in PD-related studies. However, a study on 6-OHDA rats showed that the firing rate of FSIs is increased in PD condition compared with control condition [43]. Furthermore, another group of cells, referred to as the tonically active neurons (TANs), was the only cell population that showed decreased firing rates due to DA depletion [43]. Understanding the contribution of these types of striatal cells in the appearance and stabilization of pathological dynamics in PD condition remains to be carefully and rigorously examined.

### 3.3. External Globus Pallidus (GPe)

The GPe is one of the intermediate nuclei in the BG functional structure that receive GABAergic inputs from striatal D2 MSNs acting in the indirect pathway and relays them toward the STN and, then, to the BG output nuclei (i.e., GPi/SNr). In addition, the GPe is under the influence of glutamatergic input from the STN, which together play the role of central pattern generator in the heart of the BG [9, 44, 45].

Since the amount of inhibitory drive that the GPe receives from the striatal D2 MSNs in the indirect pathway is enhanced following DA depletion, one can expect that the mean firing rate of GPe neurons is reduced in PD condition, as the rate-based model predicts [11]. This may also result in the loss of intrinsic pacemaking mechanism of GPe neurons, leading to the reduced spontaneous activity or silence of a proportion of GPe neurons, which may be correlated with the motor symptoms in PD.

Reduced mean firing rates of GPe neurons during parkinsonism were observed in MPTP-treated nonhuman primates [19, 46, 47] and parkinsonian rodents [8, 12], as well as in PD patients [48]. For instance, the mean firing rate of GPe neurons was suppressed in parkinsonian rats (14.6 ± 0.4 Hz) in comparison with the control condition (33.7 ± 1.3 Hz) [8]. The firing rate of GPe neurons was relatively decreased in another study on 6-OHDA-treated rats (26.2 ± 10.2 Hz, PD, vs. 29.3 ± 12.2 Hz, ctrl) [12]. Suppression of the mean firing rate of GPe neurons was also demonstrated in a study on 6-OHDA-treated mice so that the mean firing rate of GPe neurons was 18.9 ± 0.87 Hz in PD condition compared with 24.5 ± 1.14 Hz in the control condition [30]. In MPTP-treated monkeys, a similar descending trend was observed in the mean firing rate of GPe neurons, i.e., 76.0 ± 28.0 Hz, PD, vs. 51.0 ± 27.0 Hz, ctrl [31] (also see Table 1, row 4, and Figure 2(d)).

Interestingly, GPe neurons in animal models of PD may show changes in the neuronal firing patterns (e.g., spiking vs. bursting properties or firing irregularity) following DA depletion, rather than merely altered firing rates [12, 15, 16]. For instance, the fraction of spikes in bursts and pausing periods in the activity of GPe neurons were both elevated in PD condition in comparison with the control condition in 6-OHDA-treated rats, i.e., bursts: 44.3 ± 15.6%, PD, vs. 13.1 ± 14.2%, ctrl; and pausing: 23.2 ± 5.2%, PD, vs. 11.9 ± 4.1%, ctrl [12]. Further detailed experiments are required to reveal the critical role of neuronal firing patterns in the emergence of pathological dynamics following DA loss in PD condition.

In addition, DA depletion significantly changes synchronized activity between pairs of GPe neurons in experimental models. For instance, the activity of pairs of GPe neurons is typically not correlated in control rats and only 17.6% of pairs were significantly coherent [8]. However, in 6-OHDA-lesioned rats, most pairs of the GPe neurons were synchronized in an oscillatory manner such that 67.1% of GPe pairs were significantly coherent [8]. In healthy monkeys, only 11% of GPe cells showed significant low beta band (8–15 Hz) oscillations, whereas MPTP-treated monkeys were characterized with 39% of GPe cells developing significant oscillations [49].

### 3.4. Subthalamic Nucleus (STN)

The role of STN in shaping the normal/abnormal dynamics in the BG may be more critical than the other nuclei, since it receives inputs from the cortex (via the hyperdirect pathway) and GPe (via the indirect pathway) and relays them toward the GPi/SNr (also see Figure 1(a)). Due to its importance, the STN has been nominated as the typical target of DBS for the treatment of PD, as demonstrated in several computational and experimental studies [21–23].

Due to the suppression of inhibitory input from the GPe following DA depletion, the firing rates of STN cells should be increased in the PD state compared with the normal condition. In fact, the enhanced firing rate of STN neurons is a hallmark of PD, which has been observed in several experimental studies [9, 50]. In particular, in monkeys with MPTP-induced parkinsonism, the mean firing rate of STN neurons increases from 18.8 ± 10.3 Hz in control condition to 25.8 ± 14.9 Hz in PD state [32]. In 6-OHDA-lesioned rats, the mean firing rate of STN cells was significantly increased (34.0 ± 3.4 Hz) compared with control condition (13.8 ± 2.7 Hz) [10]. Another study on 6-OHDA-treated rats showed a qualitatively similar increasing trend for the mean firing rates of STN (17.1 ± 1.0 Hz, PD, vs. 11.8 ± 0.70 Hz, ctrl) [16] (also see Table 1, row 5, and Figure 2(e)).

It has been argued that STN neurons in both nonhuman primates and PD patients with oscillatory activity have a higher spontaneous discharge rate than those neurons that do not show oscillatory activity [32, 51]. For instance, in PD patients, the mean firing rate of STN cells that displayed oscillatory activity was approximately 53.4 Hz, whereas the mean firing rate of those STN neurons with no oscillatory discharge was approximately 43.1 Hz [51]. This observation can maximize the oscillatory frequency that is supposed to be transmitted by the neuronal spike trains and may occur in a self-organized manner in PD condition.

In addition to the mean firing rate changes of STN cells following experimental DA depletion, changes in the neuronal activity pattern of STN neurons have also been verified in several studies. Similar to GPe cells, the firing activity of STN neurons is characterized with increased bursting both in animal models of PD and human subjects [32, 46, 51, 52]. For instance, the proportion of spikes in bursts was increased and a greater proportion of neurons were characterized with an irregular discharge pattern. Increased bursting of STN cells is a characteristic of PD and may be related to the severity of PD motor symptoms [53].

Moreover, oscillatory activity in the STN has been observed in several single-unit studies of parkinsonian animals. The emergence of the synchronized activity is somehow correlated with the single-cell oscillations during parkinsonism. Studies in monkeys revealed that the MPTP treatment may not significantly change the synchronization of neuronal activity between STN cells [32, 54]. Approximately 11% of the recorded STN pairs showed synchronized activity in healthy monkeys, whereas cross-correlograms of approximately 12% of simultaneously recorded STN neurons exhibited synchronous activity after MPTP treatment [32, 54].

### 3.5. Substantia Nigra Pars Reticulata (SNr)

The SNr is one of the two BG output nuclei that receive GABAergic inputs directly from the striatum and glutamatergic inputs from the STN and projects to the thalamus (see Figure 1(a)). Following DA depletion in experimental PD models, the STN is characterized with excessive neuronal synchronization and increased firing rates of cells [10]. This suggests that the mean firing rates of SNr neurons should be relatively enhanced in PD condition in comparison with the healthy state, as addressed in a few experimental studies [33, 46].

In particular, a study on normal rats showed that the spontaneous activity of SNr cells is characterized with a tonic regular or relatively irregular discharge pattern with a mean firing rate of 15.2 ± 6.1 Hz [33]. However, the discharge pattern of SNr cells in rats with 6-OHDA SNr lesions was irregular with a mean firing rate of 21.3 ± 7.4 Hz (also see Table 1, row 6, and Figure 2(f)) [33]. Interestingly, in rats with GP lesions, both the discharge pattern of SNr cells and their mean firing rate (15.5 ± 7.5 Hz) were similar to those recorded in normal rats.

Notably, STN high-frequency stimulation (130 Hz) induced a significant decrease in mean firing rates of SNr cells in all three conditions. In normal rats, the firing rates of a large proportion of SNr cells (∼94%) were decreased in response to STN stimulation. In rats with 6-OHDA SNr lesions, this percentage was approximately 90%, and in rats with lesions of GP, it was approximately 80% [33]. These findings indicate that STN high-frequency stimulation inhibits the glutamatergic output from STN toward SNr, leading to the deactivation of SNr cells [33].

### 3.6. Internal Globus Pallidus (GPi)

The GPi is another BG output nucleus that shares similar inputs/outputs with the SNr. As previously argued in the case of SNr, the firing rates of GPi cells should also be increased in PD condition in comparison with the normal state due to the enhancement of the excitatory inputs received from the STN. This was demonstrated in a number of studies.

A study on MPTP-treated monkeys showed that the mean firing rate of GPi cells is significantly increased in PD condition in comparison with the control condition (77.9 ± 26.4, PD, vs. 57.3 ± 17.3, ctrl) [35]. The increase of mean firing rates and bursting activity of the BG output nuclei, i.e, GPi/SNr, leads to the increased inhibitory drive that is subsequently transmitted to the thalamocortical circuits and may contribute to the symptomatic expression of PD. However, in a study on 6-OHDA rats, no significant change of the mean firing rates of GPi neurons were observed in the lesioned rats compared with the control condition (29.3 ± 11.3, PD, vs. 28.0 ± 9.1, ctrl) [34] (also see Table 1, row 7, and Figure 2(g)).

Notably, experimental observations revealed that the firing pattern of GPi cells was also changed in the PD state versus the normal condition both in MPTP-treated monkeys and 6-OHDA rats. The activity pattern of GPi neurons shifts from tonic discharge in the normal state to bursting in the DA-depleted state in MPTP-treated monkeys [54, 55]. Specifically, the number of bursting neurons increased from 28.5% in the normal state to 44.1% in MPTP-treated monkeys [35]. In 6-OHDA-lesioned rats, the bursting index was also significantly increased (3.9 ± 4.3, PD, vs. 33.4 ± 24.9, ctrl) [34].

Studies in healthy monkeys showed that only 3% of the GPi cells exhibited pronounced low beta band (8–15 Hz) oscillations [49]. However, MPTP treatment significantly increased this percentage so that 43% of the GPi cells developed low beta oscillations. Cross-correlation analysis of the GPe and GPi neurons together showed weak correlated activity between the pallidal neurons in normal monkeys such that 95.6% of these pairs were not correlated [49]. After MPTP treatment, 40% of the pallidal pairs showed pronounced synchronous oscillations [49].

In summary, as listed in Table 1, the mean firing rates of neurons in the primary motor cortex show no significant change in MPTP-treated monkeys compared with control condition. This is also correct for those primary motor cortical neurons projecting to the striatum. In 6-OHDA-treated rats/mice, striatal D1R MSNs show suppressed firing rates, whereas D2R MSNs typically fire at higher rates in comparison with control condition. Some studies, however, reported no specific change in the firing rates of D2R MSNs in PD condition. Findings of experimental PD models revealed that the mean firing rate of GPe neurons is decreased both in 6-OHDA-treated rats/mice and MPTP-treated monkeys with respect to normal condition, but STN neurons are characterized with increased firing rates following DA depletion. Finally, the mean firing rate of SNr and GPi is increased in 6-OHDA-treated rats/mice or MPTP-treated monkeys compared with normal state.

## 4. Abnormal Beta Oscillations in the Corticobasal Ganglia Circuits

DA loss in PD condition can lead to alterations in the firing rates of neurons, increased neuronal synchrony, and exaggerated beta band (15–30 Hz) oscillations in the entire corticobasal ganglia circuits [10, 42]. However, how neuronal firing rate changes are related to the LFP-recorded abnormal beta oscillations is poorly understood. Two major hypotheses can be brought forward to address the origin of abnormal beta oscillations in the parkinsonian cortico-BG circuits: first, the GPe-STN microcircuit is intrinsically a center for pattern generation in the BG and can generate abnormally synchronized beta oscillations in the PD state that are subsequently spread in the entire cortico-BG loop, as experimentally supported by several observations in the GPe-STN network [8, 9, 44]; and second, the GPe-STN network does not work alone and there are extrinsic origins that can contribute to the abnormal rhythmogenesis in the parkinsonian state. Particularly, the second hypothesis emphasizes the necessity of cortical inputs to the GPe-STN network for generating the exaggerated beta oscillations in PD condition. Similarly, there are experimental studies to support this hypothesis [26, 41, 56]. In what follows we will review experimental evidence on the emergence of abnormal oscillatory activity observed in the LFP recordings of the corticobasal ganglia circuits in an attempt to track down the origin of abnormal beta oscillations in PD condition.

Excessive beta oscillations were observed in the recordings of almost all corticobasal ganglia circuits both in animal PD models and PD patients [8–10, 24, 26, 57]. Patients with PD suffer from slowed movements or troubled movement initiation that is correlated with the emergence of exaggerated beta oscillations. Abnormal beta oscillations in the motor cortex have been linked to movement-related tasks in a number of experimental studies [27, 58]. However, how exactly they are correlated with population neuronal activity and specific behavioral manifestations has remained poorly understood. It has been suggested that enhanced beta oscillations in the motor cortex of nonhuman primates modulate population neuronal activity such that the movement onset is delayed [58]. In fact, excessive beta oscillations in the motor cortex are more pronounced in MPTP-treated monkeys than the control group, resulting in reduced specificity for movement tasks [27, 36]. This may increase the movement-related coherency in neuronal activity between cortex and STN as shown both in animal PD models and PD patients [9, 24, 59–62].

The striatum is massively under the influence of DA released from the DAergic neurons in the SNc and, hence, is among first regions affected by DA loss. This suggests that dysfunction of neuronal dynamics in the striatum may be one of the possible origins of the emergence of pathological beta oscillations in PD condition [63]. Indeed, the loss of glutamatergic synapses that arise from the cortex and project onto the striatal MSNs [4] is accompanied with the weakening of synapses between MSNs [5] that may result in the abnormally enhanced synaptic transmission toward downstream networks via the indirect pathway.

A number of studies investigated PD-related changes in oscillatory activity of LFP recordings in the striatum. DA depletion can lead to the changes in the beta power of striatal LFP oscillations so that it is significantly increased in the parkinsonian mice [64]. These changes in the beta power can be reversed by administration of l-3,4-dihydroxyphenylalanine (L-DOPA) [64]. Another study in mice showed that concurrent blockade of D1Rs and D2Rs can increase the power of beta oscillations such that a significant percentage of neurons are entrained to LFP [42]. However, recordings of the striatum in MPTP-treated monkeys showed that abnormal low beta (8–15 Hz) spiking and LFP oscillations can emerge across the BG circuits including STN, GPe, and SNr, but not striatal MSNs [47]. Although no significant oscillatory activity was recorded from MSNs in the MPTP-treated condition, recordings from cholinergic interneurons of the striatum showed peaks around 10 Hz in their power spectral densities [47]. The spiking activity of STN, striatal cholinergic interneurons, and BG downstream networks was entrained by these LFP beta oscillations, but the spiking activity of striatal MSNs was unaffected [47]. Ultimately, it has been argued that DA depletion may impact on the striatal LFP oscillations in a task-dependent manner [65], likely affected by the oscillatory activity in the cortex, as suggested by concurrent LFP and electroencephalography (EEG) recordings in the striatum [66, 67].

In the GPe, the activity of neurons is normally uncorrelated but shows phase-locked, overly synchronized oscillations in MPTP-treated monkeys [68]. Qualitatively similar increased synchrony of beta oscillations was also observed in DA-depleted rodent models of PD [8, 12]. Particularly, GPe neurons in parkinsonian rats showed approximately 44.2% firing coherency at beta frequency, whereas the coherency of GPe neurons was approximately 0.6% in the control condition [8]. Notably, despite the suppression of the mean firing rate of GPe neurons in PD condition, abnormal synchronization of beta oscillations is significantly increased (nearly 100-fold compared with the intact rats) [8]. The emergence of pathological beta oscillations in the GPe of rodents has been attributed to the changes of connectivity within and between GPe and STN nuclei [69].

The role of GPe in the emergence of abnormal beta oscillations is crucial. DA depletion can lead to the increased neuronal synchrony and enhanced beta power in GPe, leading to synchronous pauses in the activity of striatal FSIs via the pallidostriatal pathway, as shown both in experimental and computational PD models [12, 30]. This enables MSNs to fire in the beta range, in this way, shaping striatal output during periods of synchronous activity in GPe [30]. In fact, the effect of phasic striatal inhibition on slow oscillations in GPe is stronger than STN excitation [70]. STN beta activity could act as the synchronizer of GPe, where the amplification of beta oscillations in the GPe-FSI-MSN loop further reinforces beta activity in the STN [30]. These abnormal beta oscillations are then propagated to the cortex and other BG nuclei. In addition, the motor cortex is another potential synchronizer of GPe activity via the corticopallidal projection [71] that bypasses the traditional direct, indirect, and hyperdirect pathways.

The emergence of abnormal neuronal oscillations in the STN is widely reported in the LFP recordings of parkinsonian animals and humans. Synchronized beta oscillations at high frequencies (15–30 Hz) were observed in most STN neurons of PD patients with tremor at rest, which were suppressed by voluntary movements or DA medication [24]. However, abnormally synchronized beta oscillations were also present in patients with no noticeable tremor [24, 59, 72]. The number of neurons with oscillatory activity is notably increased in MPTP-treated monkeys, particularly in the low beta (8–15 Hz) frequency ranges [32, 46]. Similarly, in 6-OHDA-lesioned rats, DA depletion increases both the power and coherency of high-frequency (20–30 Hz) beta oscillations observed in the LFP recordings from the STN [10, 26].

To shed light on the origin of the subthalamic LFP beta oscillations and to reveal that to what extent they are related to local neuronal discharges, Kühn and colleagues recorded both LFPs and multi-neuronal activity from microelectrodes implanted into the STN of PD patients [61]. They noticed a significant increase in beta band LFP activity when microelectrodes inserted in the STN, suggesting that beta oscillations are generated within the STN. These observations suggest that subthalamic neuronal discharges are locked to LFP beta oscillations, implying that LFP activity is a measure of synchronous activity in STN neuronal populations [61]. Another study on PD patients revealed significant coherency in LFP in the beta frequency, where approximately 90% of neurons had beta oscillatory activity within the STN [14]. These results suggest that generators of LFP beta oscillations are possibly located within the STN.

LFP recording from the rat STN [10] showed results that were similar to those obtained in PD patients, implying that the generation of beta LFPs may take place within the STN. Despite experimental evidence that the STN is intrinsically a pacemaker [73], typical unit activities recorded during beta oscillations were not observed ex vivo [8], suggesting that in addition to the intrinsic membrane properties of STN neurons, intact afferents are necessary for the generation of overly synchronized outputs from the STN [8, 10]. Hence, the emergence and stabilization of abnormal beta oscillations in the parkinsonian cortico-BG circuits may be the result of the cooperation of several networks, not only one nucleus.

As revealed by the findings of DBS studies, the GPe and STN are mediated by a largely overlapped common functional network [74], in which together shape the center of normal/abnormal rhythmogenesis in the BG, as implicated in several computational [45, 75] and experimental [9, 75] studies. The activity of GPe neurons and the LFP of STN may show increased coherence in animal PD models [8, 26], due to the presence of reciprocal interactions between GPe and STN. In addition, it has been argued that the GPe-STN network forms a feedback microcircuit that is involved in synchronized bursting such that both GPe and STN can spontaneously produce synchronized oscillating bursts [44]. Although the bursts of GPe are weaker than the STN bursts, they are necessary for the generation of oscillatory bursts by inducing rebound excitations within the STN [44]. These observations encouraged numerous studies to focus on the role of GPe-STN network in the generation of parkinsonian beta oscillations.

The role of the GPe-STN microcircuit in pattern generation can be considered both intrinsically and extrinsically. Intrinsically, the GPe and STN form an excitatory-inhibitory network, which inherently is a classical pacemaker. This was scrutinized both computationally [45, 75] and experimentally [9, 44]. Conditions required for the generation of beta oscillations in the GPe-STN network, e.g., changes in synaptic connectivity within and between GPe and STN or imbalance of excitatory-inhibitory input, were addressed in modeling studies [45]. Experimentally, an accurate study on the discharges of GPe and STN neurons in parkinsonian rats showed rhythmic sequences of recurrent excitation and inhibition in the GPe-STN network and lateral inhibition between GPe neurons that can underlie the generation of abnormal beta oscillations in the parkinsonian GPe-STN network and entire cortico-BG circuits [8].

Extrinsically, cortical inputs can shape the rhythmic activity in the GPe-STN network. A number of studies have shown that the activity of STN neurons is shaped by GPe in response to cortical inputs, suggesting that the interaction between GPe and STN is modulated by cortical inputs that cause rhythmic oscillatory activity in the STN-GPe network [76, 77]. STN LFP oscillations and EEG recordings from cortex show significant coherence in the beta frequency in 6-OHDA-lesioned rats [10, 26]. Moreover, synchrony in the beta frequency band is notably increased between GPe and STN, and between STN and cortex following DA depletion [8, 26]. Finally, following DA depletion in 6-OHDA-lesioned rats, both GPe and STN exhibit low-frequency oscillatory activity, but both the amplitude and period of cortical slow-wave activity are relatively unaffected by DA depletion [56]. These observations suggest that the GPe and STN may be more sensitive to the cortical rhythms in the DA-depleted state.

Abnormal beta oscillations are finally propagated to the BG output nuclei (i.e., GPi/SNr). LFP recordings from the SNr of parkinsonian rats showed that power of beta activity in the DA-lesioned hemisphere is significantly greater than the intact (nonlesioned) hemisphere during rest [26, 78, 79]. Similar to those findings obtained in other BG nuclei, active movement suppressed the beta oscillatory activity of SNr [26, 78, 79]. Furthermore, the SNr spiking activity was more synchronized with the beta oscillations in the DA-lesioned hemisphere compared with the nonlesioned hemisphere [78, 79], where the beta activity between cortex and SNr was notably coherent [80]. In addition, studies on MPTP-treated monkeys demonstrated that in normal state only 5% of GPi neurons show oscillatory activity [32]. However, the proportion of neurons characterized with strongly synchronized beta oscillatory activity increased to 25.3% in the DA-depleted state [32]. Another study on MPTP-treated monkeys reported that only 3% of GPi neurons have oscillatory activity in the normal state, whereas 43% of GPi neurons showed significant beta oscillatory activity in PD condition [49].

## 5. Other Possible Mechanisms

The emergence of abnormal beta oscillations in PD condition cannot be solely attributed to the changes of neuronal dynamics following DA loss. Other complex mechanisms may be involved. For instance, gradual appearance and stabilization of abnormal beta oscillations and associated motor symptoms following DA depletion suggest that plasticity mechanisms at different levels are involved in the emergence of pathological activity-connectivity states within the BG. At the structural level, DA depletion may lead to the morphological changes, i.e., creation of new synapses and elimination of existing ones due to structural plasticity [81, 82]. At the functional level, the strength of synapses is regulated by DA-mediated synaptic plasticity mechanisms according to the activity of neurons [6, 83]. These structural and functional changes in synaptic connectivity within and between different BG nuclei may contribute to the emergence and stabilization of pathological neuronal activity during parkinsonism.

Impaired DA-mediated synaptic plasticity [84, 85] is involved in several neuropsychiatric disorders such PD, drug addiction, schizophrenia, attention-deficit/hyperactivity disorder (ADHD), and obsessive-compulsive disorder (OCD) [83, 86]. Spike-timing-dependent plasticity (STDP) is a type of synaptic plasticity that modifies the synaptic strengths between neurons based on the correlation of pre- and postsynaptic spikes [87–89]. The findings of experimental studies revealed that DA loss can reshape the temporal resolution of the STDP profile required for the induction of long-term potentiation (LTP) and long-term depression (LTD) [84, 85, 90, 91]. For example, DA loss can modulate the amplitude of LTP and LTD, convert LTD to LTP, and increase the threshold required for LTP/LTD induction [84, 85, 90]. This can lead to the pathological synaptic transmission and abnormal activity-dependent modification of the synaptic connectivity within the BG [6], contributing to the emergence of pathological activity-connectivity states, which is a hallmark of PD [9, 50].

In addition, STDP can shape multistable network dynamics [92, 93], computationally ascribed to normal and diseased basins of attractions. Interestingly, multistability may not only occur on a network level but also on a single-neuron level, as illustrated in a study of firing properties of STN neurons in PD patients [94]. Such multistable dynamics (potentially mediated by STDP) can further contribute to the stabilization of PD state by abnormal reshaping of neuronal activity and synaptic connectivity, simultaneously [95, 96]. This can finally lead to the shifting of activity-connectivity patterns from physiological states (weak synchrony, weak connectivity) toward pathological states (strong synchrony, strong connectivity). In this case, external intervention such as brain stimulation therapy is needed to restore the normal condition.

However, the classical view of STDP neglects the role of neuromodulators in the learning process during behavioral tasks [97, 98]. The core of the classical STDP relies on the coactivation of neurons where merely the joint activity of pre- and postsynaptic neurons determines synaptic modification [87–89]. Motivated by the experimental observations on DA-mediated synaptic plasticity [84, 85, 90, 91], a number of modeling studies took into account the effect of neuromodulators on STDP [99–101]. The effect of DA, for instance, can be simulated by the inclusion of a third factor [99–101] to the conventional two-factor Hebbian plasticity rules, termed as neo-Hebbian synaptic plasticity [102], representing DAergic neuromodulatory signals. Such modeling approaches may shed light on the complicated effect of DA on synaptic plasticity.

## 6. Conclusion

Although the exact relationship between pathological neuronal oscillations and motor symptoms in PD is not fully understood, suppression of abnormal beta oscillations can induce therapeutic effects in PD patients. Hence, it is important to identify networks that are responsible for the generation and propagation of abnormal beta oscillations within the CBGTC loop. Due to its excitatory-inhibitory nature, the GPe-STN network within the BG is intrinsically considered as a center for the generation of abnormal beta oscillations during parkinsonism that are then propagated to the other networks in the CBGTC loop.

However, it would be naive to think that merely one or two BG nuclei are responsible for abnormal rhythmogenesis in PD condition. Here, we reviewed relevant experimental studies that took into account the effect of extrinsic cortical (direct) and striatopallidal (indirect) inputs on the GPe-STN rhythmogenesis to highlight the role of other corticosubcortical networks in the emergence and stabilization of parkinsonian beta oscillations. This review provides insights into the role of cortex and different BG nuclei in shaping abnormal beta oscillations during parkinsonism, which may enable potential target-specific interventions for the treatment of PD.

In fact, the role of cortex and its inputs to the GPe-STN network is critical. The STN receives cortical inputs directly via the hyperdirect pathway and indirectly from the indirect pathway mediated by the striatopallidal pathway. These pathways can be assumed as the origin of generation and reinforcement of abnormal beta oscillations in PD condition. Moreover, pathological neuronal activity may be accompanied with pathological synaptic connectivity. In fact, competition between excitatory and inhibitory inputs from the hyperdirect and indirect pathways following DA loss may trigger abnormal plasticity mechanisms that further support the emergence of pathological activity-connectivity patterns. Nonetheless, the question that which structure(s) triggers the exaggerated beta oscillations cannot be answered so simply since the CBGTC loop is characterized with several strongly interconnected and highly overlapped networks that compete or cooperate to determine the dynamics and structure in physiological and pathological conditions.

## Figures and Tables

**Table 1:** Changes of the neuronal mean firing rates within the cortico-BG circuits observed in 6-OHDA rats/mice or MPTP monkeys in control (normal) and parkinsonian condition.

Mean firing rate ± SD (Hz)					
No.	Nuclei	Control	PD	Animal model	Reference

1	Cortex	5.0 ± 0.8	4.5 ± 0.5	MPTP monkey	Goldberg et al. [27]
2	D1 MSN	1.61 ± 0.19	0.11 ± 0.04	6-OHDA mouse	Ryan et al. [29]
3	D2 MSN	1.42 ± 0.28	1.24 ± 0.23	6-OHDA mouse	Ryan et al. [29]
		2.1 ± 1.2	6.4 ± 2.7	6-OHDA rat	Kita and Kita [12]

4	GPe	33.7 ± 1.3	14.6 ± 0.4	6-OHDA rat	Mallet et al. [8]
		29.3 ± 12.2	26.2 ± 10.2	6-OHDA rat	Kita and Kita [12]
		24.5 ± 1.14	18.9 ± 0.87	6-OHDA mouse	Corbit et al. [30]
		76.0 ± 28.0	51.0 ± 27.0	MPTP monkey	Filion and Tremblay [31]

5	STN	18.8 ± 10.3	25.8 ± 14.9	MPTP monkey	Bergman et al. [32]
		13.8 ± 2.7	34.0 ± 3.4	6-OHDA rat	Mallet et al. [10]
		11.8 ± 0.7	17.1 ± 1.0	6-OHDA rat	Breit et al. [16]

6	SNr	15.2 ± 6.1	21.3 ± 7.4	6-OHDA rat	Benazzouz et al. [33]
7	GPi	28.0 ± 9.1	29.3 ± 11.3	6-OHDA rat	Kita and Kita [34]
		57.3 ± 17.3	77.9 ± 26.4	MPTP monkey	Leblois et al. [35]

## Data Availability

All data generated or analyzed during this study are included in this published article.
